# Predictive value of DCE-MRI and IVIM-DWI in osteosarcoma patients with neoadjuvant chemotherapy

**DOI:** 10.3389/fonc.2022.967450

**Published:** 2022-10-14

**Authors:** Xibin Xia, Lu Wen, Feng Zhou, Junjun Li, Qiang Lu, Jun Liu, Xiaoping Yu

**Affiliations:** ^1^ Department of Diagnostic Radiology, Hunan Cancer Hospital and The Affiliated Cancer Hospital of Xiangya School of Medicine, Central South University, Changsha, China; ^2^ Department of Orthopedics and Soft Tissue, Hunan Cancer Hospital and the Affiliated Cancer Hospital of Xiangya School of Medicine, Central South University, Changsha, China; ^3^ Department of Pathology, Hunan Cancer Hospital and The Affiliated Cancer Hospital of Xiangya School of Medicine, Central South University, Changsha, China

**Keywords:** osteosarcoma, DCE-MRI, IVIM-DWI, clinical outcomes, predictor

## Abstract

**Objective:**

To investigate the predictive value of dynamic contrast enhanced MRI (DCE-MRI) and intravoxel incoherent motion diffusion-weighted imaging (IVIM-DWI) for clinical outcomes of osteosarcoma patients with neoadjuvant chemotherapy.

**Methods:**

The present prospective single-arm cohort study enrolled 163 patients of osteosarcoma during July 2017 to July 2022. All patients received the same treatment strategy of neoadjuvant chemotherapy. Both DCE-MRI and IVIM-DWI were conducted for the patients before the chemotherapy, as well as after one or two chemotherapy treatment cycles. The imaging parameters of contrast agent transfer rate between blood and tissue (*K^trans^*), contrast agent back-flux rate constant (*K_ep_*), extravascular extracellular fractional volume (*V_e_*), as well as pure diffusion coefficient (*D* value), pseudo-diffusion coefficient (D* value), apparent diffusion coefficient (*ADC*) and the perfusion fraction (*f* value) were recorded. RECIST standard [complete response (CR), partial response (PR), stable disease (SD), progressive disease (PD)] was used as the main clinical outcome.

**Results:**

After two treatment cycles, 112 (68.71%) cases were with CR and PR, 31 (19.02%) cases were with SD and 20 cases (12.27%) were with PD. After 1~2 treatment cycles, patients with CR/PR showed significantly markedly lower *K^trans^*, *K_ep_*, *V_e_* values, while higher *D*, *ADC* and *f* values compared with SD or PD patients. Alkaline phosphatase (ALP) and lactate dehydrogenase (LDH) were positively correlated with values of *K^trans^*, *K_ep_*, and *V_e_*, while negative correlation was observed between ALP and values of *D*, *ADC* and *f*, as well as between LDH and *D* and *ADC* after the whole treatment. *D* and *K_ep_* values after two treatment cycles showed the best predictive value for diagnosis of PD. The values of *K^tran^*, *K_ep_*, *ADC* as well as ALP and LDH were all risk factors for PD after neoadjuvant chemotherapy.

**Conclusion:**

DCE-MRI and IVIM-DWI have the potential to predict clinical outcomes of osteosarcoma patients with neoadjuvant chemotherapy.

## Introduction

According to a recent report, osteosarcoma accounts for about 2.4% tumors in children, with an incidence around 2~5 per million in all population worldwide (1–3). Generally, surgery combined with chemotherapy is the main treatment method for osteosarcoma patients (4, 5). For patients without metastasis, neoadjuvant chemotherapy, also known as preoperative chemotherapy, is usually used to decline the tumor size and adjust the patients’ condition for further surgery (6, 7). It has been reported that neoadjuvant chemotherapy could also enhance the prognosis of osteosarcoma patients (8). Deng et al. found neoadjuvant chemotherapy could regulate the tumor immunologic microenvironment in osteosarcoma (9). Another study demonstrated that preoperative neoadjuvant chemotherapy improved overall survival rate and prolonged disease-free survival of osteosarcoma (10). During the treatment of neoadjuvant chemotherapy, the early prediction of clinical efficacy is of great significance (11, 12). Previous studies demonstrated the potential use of T1 or T2-weighted imaging (T1WI or T2WI) in magnetic resonance imaging (MRI) for predicting the response of neoadjuvant chemotherapy. It was found that T2-weighted fat-saturated and contrast-enhanced T1-weighted images could be used for differential diagnosis of osteosarcoma and Ewing sarcoma (13). Another study showed that chemotherapy response could be predicted by T2WI with AUC=0.70 by using MRI-based statistical texture analysis in osteosarcoma (14). In other cancer types, such as breast cancer, it was also found that T1WI might be associated with the chemotherapy response, in which baseline contrast and entropy values on the 1-, 2-, and 3-minute postgadolinium T1WI might be different in patients with different chemotherapy response (15, 16). However, to accurately predict the efficacy of neoadjuvant chemotherapy is still a clinical challenge.

Dynamic contrast enhanced magnetic resonance imaging (DCE-MRI) is a newly developed method for MRI, which shows well diagnostic value for many cancers, such as rectal cancer, gastric cancer and lung cancer (17–19). Except for DCE-MRI, intravoxel incoherent motion diffusion-weighted imaging (IVIM-DWI), which has the ability to separate pure diffusion movement and perfusion, shows better efficacy than the traditional MRI or apparent diffusion coefficient (ADC) map from DWI (20, 21). However, up to now, few studies focused on the predictive value of DCE-MRI and IVIM-DWI for clinical outcomes of osteosarcoma patients with neoadjuvant chemotherapy.

In the present study, we aimed to conduct a prospective single-arm cohort study to investigate the predictive value of DCE-MRI and IVIM-DWI for clinical outcomes of osteosarcoma patients with neoadjuvant chemotherapy. This study might provide more clinical evidence for application of DCE-MRI and IVIM-DWI in osteosarcoma patients.

## Methods and materials

### Patients and treatment

The present prospective single-arm cohort study enrolled 163 patients of osteosarcoma who came to our hospital during July 2017 to July 2022. The inclusion criteria were: 1) the diagnosis of osteosarcoma was all confirmed by histological analysis and patients were diagnosed as osteosarcoma for the first time; 2) all patients received neoadjuvant chemotherapy during the study period. The following patients were excluded: 1) patients who received chemotherapy, radiotherapy or targeting therapy before the study; 2) pregnant patients; 3) patients with distant metastasis. All patients meeting the inclusion criteria were consecutively recruit. The written informed consent was obtained from all patients. The present study was approved by the Ethical Committee of Hunan Cancer Hospital and the Affiliated Cancer Hospital of Xiangya School of Medicine, Central South University.

All patients received the same treatment strategy of neoadjuvant chemotherapy with adriamycin (ADR) + cisplatin (DDP) + methotrexate (MTX) + ifosfamide (IFO) before the surgery. Each treatment cycle included ADR 60 mg/m^2^ in two days, DDP 100 mg/m^2^, MTX 8~12 g/m^2^, and IFO 2 g/m^2^ for 5 days. One treatment cycle lasted for 3 weeks. The chemotherapy stopped for 2 weeks after one cycle of treatment. The whole chemotherapy included two-treatment cycles in 10 weeks before the surgery.

### Imaging measurement of DCE-MRI and IVIM-DWI

All patients received both DCE-MRI and IVIM-DWI before the chemotherapy, as well after one cycle and two cycles of the neoadjuvant chemotherapy. The conventional MRI was conducted using a 1.5-Tesla MRI scanner (Optima MR360, GE Healthcare) as described elsewhere (22).

For DCE-MRI, patients received 1) LAVA-T1WI (Flip Angle 2° and 15°, TR 3 ms, TE 1.3 ms, FOV 240 cm, layer thickness 3 mm and layer spacing 0.5 mm), 2) LAVA-T1WI dynamic enhanced scanning with 56 phases, 6 s/phase, 26 images in 5 min and 29 seconds (Flip Angle 15°, TR 3 ms, TE 1.3 ms, FOV 240 cm, layer thickness 3 mm and layer spacing 0.5 mm). After 18 s of LAVA-T1WI dynamic enhanced scanning, patients were injected with gadolinium diamine through elbow vein (0.1 mmol/kg, 2 ml/s), following with injection of 15 ml normal saline (2 ml/s). The data was analyzed using Cinetool package (GE Healthcare). The parameters of contrast agent transfer rate between blood and tissue (*K^trans^*), contrast agent back-flux rate constant (*K_ep_*), extravascular extracellular fractional volume (*V_e_*) were recorded.

For IVIM-DWI, patients received single-shot echo-planar imaging (SE-DW-EPI) sequence for cross-sectional imaging with 13 *b* values (0, 10, 20, 30, 50, 80, 100, 150, 200, 400, 600, 800 and 1000 s/mm^2^). The parameters were TR 4225 ms, TE 97 ms, FOV 240 cm, layer thickness 3 mm and layer spacing 0.5 mm. Analysis of IVIM-DWI was performed by using double exponential model DWI analysis software. The following parameters were recorded: pure diffusion coefficient (*D* value), pseudo-diffusion coefficient (D* value), apparent diffusion coefficient (*ADC*) and the perfusion fraction (*f* value).

### Main clinical outcomes and data collection

In the present study, we used RECIST standard to evaluate the treatment response as described elsewhere (23). Briefly, the treatment response was defined as: 1) complete response (CR), patients with complete resolution of the target lesions; 2) partial response (PR), patients with >30% decline of target tumor’s diameter; 3) progressive disease (PD), patients with ≥20% elevation of target tumor’s diameter; 4) stable disease (SD), the target tumor’s diameter between PR and PD. Besides, histological response was defined as good (tumor necrosis rate ≥90%) or poor (tumor necrosis rate <90%).

Patients’ clinical characteristics including age, sex, TNM stage, and clinical outcomes were recorded. Serum carcinoembryonic antigen (CEA) was evaluated by enzyme linked immunosorbent assay (ELISA) using commercial kit (Abcam) before and after the whole treatment. The serum levels of alkaline phosphatase (ALP) and lactate dehydrogenase (LDH) were tested using corresponding kits purchased from Nanjing Jiancheng Bioengineering Institute, China.

### Statistical analysis

The data distribution was analyzed by Kolmogorov-Smirnov method. The measurement data was expressed as mean ± SD for normally distributed data and median (range) was used for non-normally distributed data. Comparison for continuous data were analyzed by t test (paired or unpaired) or Mann-Whitney U test. Kruskal-Wallis test or One-way analysis of variance (ANOVA) followed by Tukey’s *post hoc* test was used for comparison among three or more groups. Chi square test was used for comparing rates. Spearman’s analysis was used for correlation analysis. The ROC curve was used for evaluating the diagnostic value. Logistic regression was performed to analyze the risk factor of PD using a step back method. *p*<0.05 was defined as statistically different. All calculations were made by SPSS 18.0 and GraphPad 6.0.

## Results

### Basic clinical characteristics of all osteosarcoma patients

This study recruit 163 osteosarcoma patients. The basic clinical characteristics of all patients when admission was shown in **Table 1**. Among all patients, 69 (42.33%) cases were with TNM stage I, 53 (32.52%) cases were with TNM stage II, while 41 (25.15%) cases were with TNM stage III. After two cycles of the treatment, 112 (68.71%) cases were with CR and PR, 31 (19.02%) cases were with SD and 20 cases (12.27%) were with PD. Meanwhile, 52 (31.90%) cases showed good necrotic rate (≥90%). No statistical difference was found among patients with different clinical outcomes.

**Table 1:** Basic clinical characteristics of all patients.

Variables	All patients (n=163)	CR/PR (n=112)	SD (n=31)	PD (n=20)	*p* ^a^
Age (y)	27 (7~40)	25 (7~40)	28 (9~39)	31.5 (9~40)	0.099
Sex, male (%)	98 (60.12)	66 (58.93)	19 (61.29)	13 (65.00)	0.673
TNM stage, n (%)					0.917
I	69 (42.33)	48 (42.86)	13 (41.94)	8 (40.00)	
II	53 (32.52)	37 (33.04)	10 (32.26)	6 (30.00)	
III	41 (25.15)	27 (24.11)	8 (25.81)	6 (30.00)	
Site of tumor, n (%)					0.643
Femur	84 (51.53)	56 (50.00)	17 (54.84)	11 (55.00)	
Tibia/fibula	36 (22.09)	25 (22.32)	7 (22.58)	4 (20.00)	
Humerus	21 (12.88)	16 (14.29)	3 (9.68)	2 (10.00)	
Pelvis	12 (7.36)	9 (8.04)	2 (6.45)	1 (5.00)	
Head/neck	9 (5.52)	5 (3.07)	2 (6.45)	2 (10.00)	
Other	1 (0.61)	1 (0.89)	0 (0)	0 (0)	
Necrosis, n (%)					
Good (≥90%)	52 (31.90)	–	–	–	
Poor (<90%)	111 (68.10)	–	–	–	

a Comparison for continuous data were analyzed by Kruskal-Wallis test post hoc for comparison among three or more groups for non-normally distributed data (age). Rates were compared by Chi square test.

### Dynamic alteration of imaging parameters for osteosarcoma patients with different clinical outcomes during treatment period

**Figure 1:**
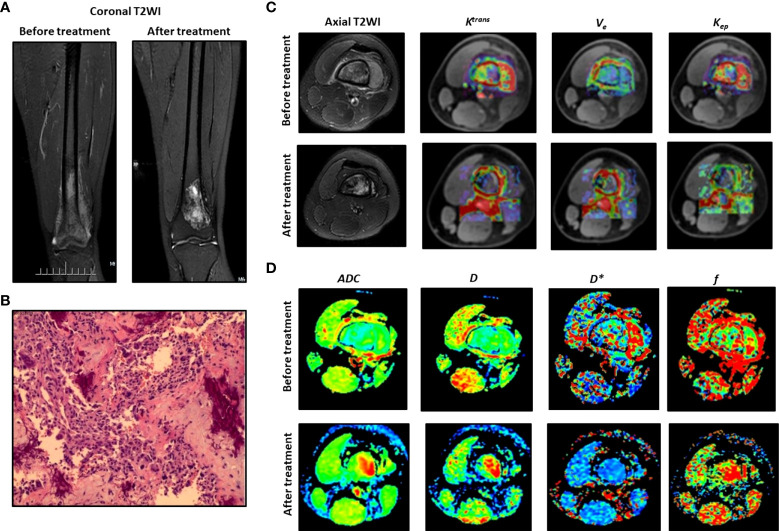
Typical DCE-MRI and IVIM-DWI images and the parameters for an 18-year-old male patient. **(A)** Coronal T2WI before and after treatment. **(B)** Histological analysis. **(C)** Axial T2WI and *K^trans^*, *K_ep_*, *V_e_*. **(D)** Images for *D*, D*, *ADC* and *f*.

**Figure 2:**
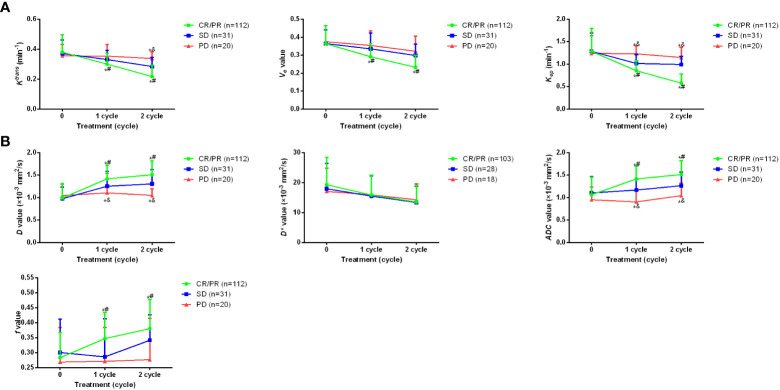
Dynamic alteration of imaging parameters for osteosarcoma patients with different clinical outcomes during treatment period. **(A)** *K^trans^*, *K_ep_*, and *V_e_* of DCE-MRI before treatment as well as after 1~2 cycles of the treatment. **(B)** changes of *D*, D*, *ADC* and *f* value of IVIM-DWI before treatment as well as after 1~2 cycles of the treatment. *p <0.05 vs PD, ^#^ *p <*0.05 vs SD, ^&^ *p <*0.05 vs CR/PR. Comparison for continuous data were analyzed by t test (paired or unpaired) or Mann-Whitney U test.

Then, the imaging parameters of *D*, D*, *ADC* and *f* value of IVIM-DWI, as well as *K^trans^*, *K_ep_*, and *V_e_* of DCE-MRI before, during and after treatment were analyzed in patients with different clinical outcomes (**Figure 1**). As shown in **Figure 2**, no statistical difference was found for all parameters before the study. For parameters of DCE-MRI, after 1~2 cycles of treatment, patients with CR/PR showed markedly lower *K^trans^*, *K_ep_*, *V_e_* values compared with the SD and PD patients (*p <*0.05). Meanwhile, *K^trans^* and *K_ep_* values were significantly elevated in PD patients compared with the SD patients (*p <*0.05). For parameters of IVIM-DWI, after 1~2 cycles of treatment, patients with CR/PR showed significantly higher values of *D*, *ADC* and *f* values compared with SD or PD patients (*p <*0.05). However, the value of D* was only remarkably lower in CR/PR patients than PD patients (*p <*0.05). All these results indicated that the alteration of imaging parameters was associated with the clinical outcomes of the osteosarcoma patients.

### Association between imaging parameters and serum tumor biomarkers

**Figure 3:**
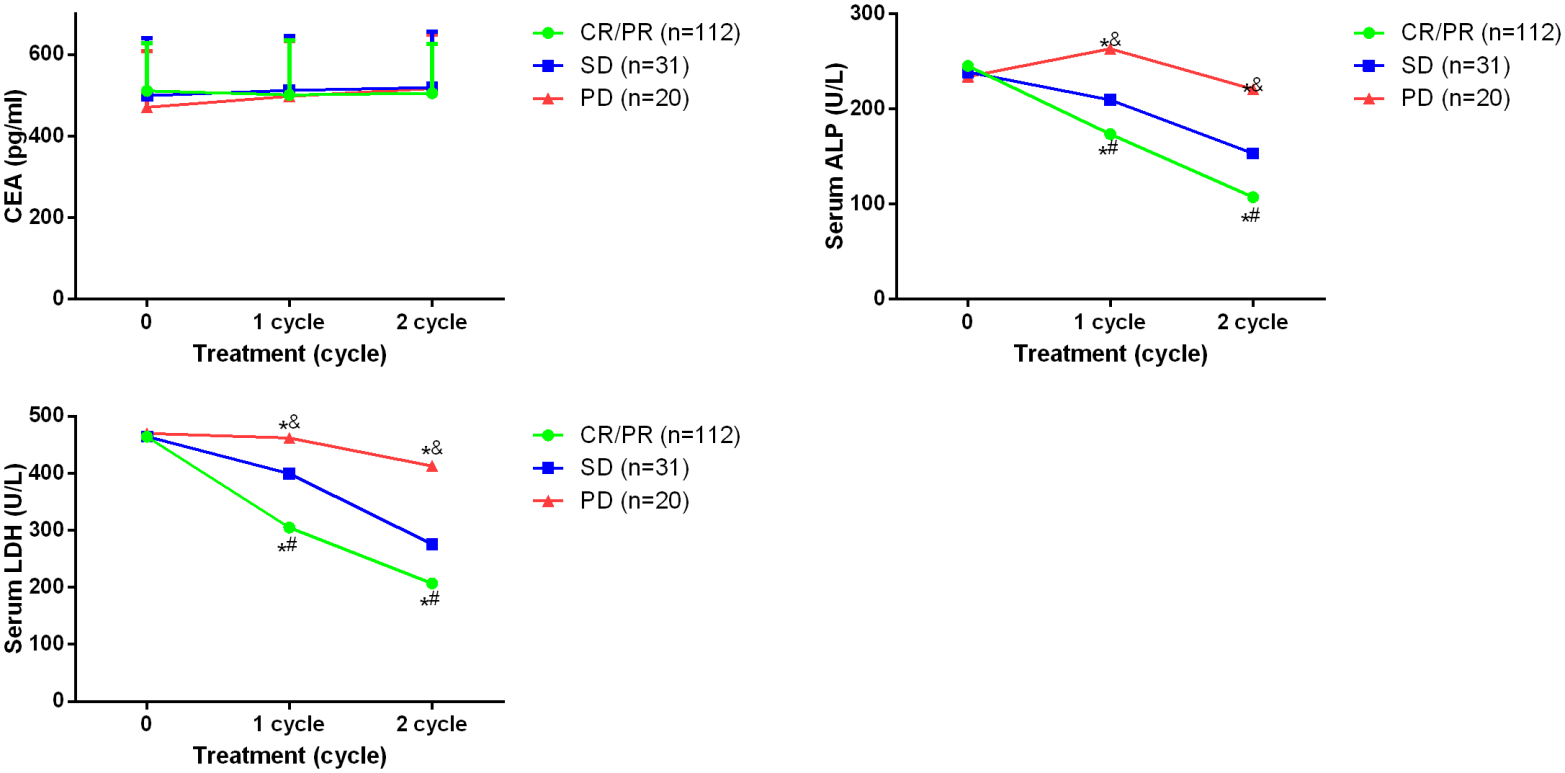
Serum levels of CEA, ALP and LDH in osteosarcoma patients before and after 2 cycles of the treatment. *p <0.05 vs PD, ^#^ *p <*0.05 vs SD, ^&^ *p <*0.05 vs CR/PR. Comparison for continuous data were analyzed by t test (paired or unpaired) or Mann-Whitney U test.

To further investigate the clinical values of the changes of parameters, the correlation between parameters and serum tumor biomarkers CEA, ALP and LDH was investigated. It was found after 2 cycles of the treatment, both ALP and LDH showed remarkably lower levels in CR/PR and SD patients compared with the baseline (**Figure 3**). However, in PD patients, the changes of ALP and LDH showed no significant difference. Besides, CEA levels didn’t alter significantly after treatment. Further Spearman’s analysis showed that ALP and LDH were positively correlated with values of *K^trans^*, *K_ep_*, and *V_e_*, while negative correlation was observed between ALP and values of *D*, *ADC* and *f*, as well as between LDH and *D* and *ADC* (all values after the whole treatment) (**Table 2**). Positive correlation was only found between CEA and *D* value for CEA.

**Table 2:** Spearman’s analysis for imaging parameters and serum tumor biomarkers after the whole treatment.

Variables	CEA		ALP		LDH	
	Spearman’s correlation	*p*	Spearman’s correlation	*p*	Spearman’s correlation	*p*
*K^trans^*	0.145	0.065	0.280	<0.001	0.338	<0.001
*K_ep_*	-0.014	0.862	0.365	<0.001	0.444	<0.001
*V_e_*	0.107	0.175	0.228	<0.001	0.321	<0.001
*D*	0.156	0.047	-0.239	0.002	-0.268	0.001
D*	0.074	0.351	-0.139	0.078	0.108	0.169
*ADC*	0.005	0.947	-0.203	0.009	-0.230	0.003
*f*	-0.137	0.080	-0.233	0.003	-0.124	0.115

### Diagnostic value of imaging parameters for osteosarcoma patients with PD after neoadjuvant chemotherapy

**Figure 4:**
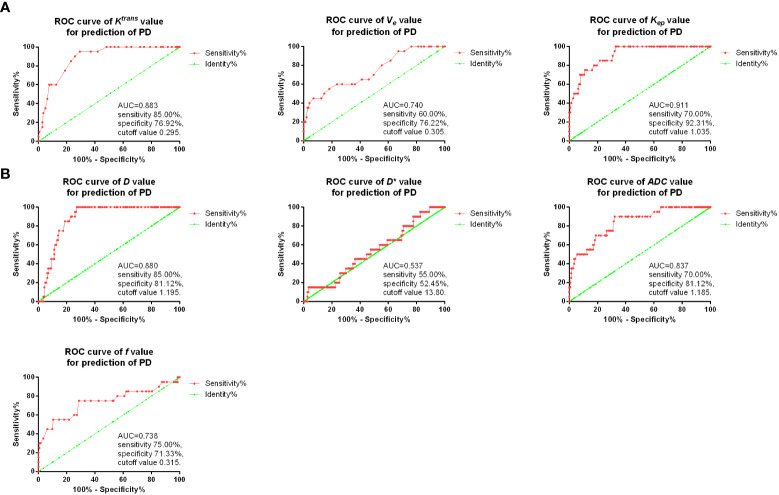
ROC curves of *K^trans^*, *K_ep_*, and *V_e_* **(A)**, as well as *D*, D*, *ADC* and *f* **(B)** in predicting PD.

Then, ROC curves were used for the diagnostic value of *D*, D*, *ADC* and *f*, as well as *K^trans^*, *K_ep_*, and *V_e_* after two cycles of the treatment for predicting PD. As shown in **Figure 4**, it was found that in DCE-MRI parameters, *K_ep_* value after two treatment cycles showed the best sensitivity 70.00% and specificity 92.31% with AUC 0.911, and cutoff value of 1.035. In IVIM-DWI parameters, *D* value after two treatment cycles showed the best sensitivity 85.00% and specificity 81.12% with AUC 0.880, and cutoff value of 1.195.

### Imaging parameters as a predictive and risk factor for PD of osteosarcoma

Binary logistic regression was then conducted for analysis of risk factor for PD of osteosarcoma after neoadjuvant chemotherapy. It was found that values of *K^trans^*, *K_ep_*, *ADC*, as well as ALP and LDH were all risk factors for PD after neoadjuvant chemotherapy (**Table 3**).

**Table 3:** Binary logistic regression for analysis of risk factor for PD of osteosarcoma after neoadjuvant chemotherapy.

Variables	Wald	Odds ratio	95% CI	*p*
Age	0.016	0.994	0.908~1.089	0.901
Sex	0.304	1.630	0.287~9.237	0.581
TNM stage	0.151	0.785	0.231~2.665	0.698
Site of tumor	0.067	0.915	0.469~1.787	0.796
*K^trans^* after treatment	8.356	1.341	1.099~1.636	0.004
*K_ep_* after treatment	7.961	1.098	1.029~1.171	0.005
*V_e_* after treatment	0.005	1.005	0.877~1.151	0.944
*D* after treatment	1.457	0.091	0.002~4.476	0.227
D* after treatment	2.268	0.845	0.678~1.052	0.132
*ADC* after treatment	4.343	0.009	0.000~0.753	0.037
*f* after treatment	3.083	0.000	0.000~2.852	0.079
CEA after treatment	0.046	0.999	0.992~1.006	0.831
ALP after treatment	14.132	1.030	1.014~1.045	<0.001
LDH after treatment	16.434	1.023	1.012~1.034	<0.001

## Discussion

Nowadays, neoadjuvant chemotherapy is widely applied before the surgery of cancer treatment, including osteosarcoma. However, it is still not easy to accurately predict the response to neoadjuvant chemotherapy. In our study, we demonstrated that both DCE-MRI and IVIM-DWI could predict the clinical outcomes of osteosarcoma patients after neoadjuvant chemotherapy.

Compared to the information that conventional T1WI or T2WI can provide, DCE-MRI can reflect microvascular distribution and blood perfusion in tumor tissues. In an early study, the authors compared the predictive value between non-enhanced MRI (T1WI and T2WI data) and DEC-MRI after neoadjuvant chemotherapy and found that DCE-MRI successfully predicted the chemotherapy response of 80% patients, while tumor volume measurements only accurately predicted response of 60% patients (24). In another work, it was found that DEC-MRI could achieve significantly better diagnostic value (AUC 0.90, sensitivity 86% and specificity 93%) than conventional T2WI (AUC 0.76, sensitivity 86% and specificity 73%) for assessing response to neoadjuvant therapy in locally advanced rectal cancer (25). Currently, DCE-MRI has been used in diagnosis or prediction of many cancers. In a meta-analysis, it was found that DCE-MRI might have high sensitivity and specificity (pooled sensitivity of 0.80 and specificity 0.84) in prediction of pathological complete response after chemotherapy in breast cancer patients (26). In a recent study, Heethuis et al. found that DCE-MRI showed good predictive efficacy for complete response (with the best sensitivity of 90% and specificity of 62.9%) in esophageal cancer patients after neoadjuvant chemoradiotherapy (27). In another research, it was found that standardized index of shape (SIS) tool could be used for analyze the DEC-MRI results in advanced rectal cancer patients, which could predict non-responders after neoadjuvant chemoradiotherapy with a sensitivity of 95.9%, specificity of 84.7% and an accuracy of 91.8% (28). In 2021, Zeng et al. demonstrated that the parameters of slope, maximum signal intensity, time to peak, signal enhanced extent, washout rate, and enhancement rate in DEC-MRI had the potential to predict the response to neoadjuvant chemotherapy in osteosarcoma patients, with the best sensitivity of 83.3% and 92.3% (29). However, except for the above research, very few studies focused on application of DCE-MRI in osteosarcoma, especially for its predictive value after neoadjuvant chemoradiotherapy. In our research, we demonstrated that osteosarcoma patients with CR/PR showed markedly lower *K^trans^*, *K_ep_* and *V_e_* values compared with the SD and PD patients, which were positively correlated with ALP and LDH. Besides, we also observed that *K_ep_* value after two treatment cycles showed good predictive efficacy for PD in osteosarcoma patients.

Compared with other MRI methods such as diffusion-weighted MRI and DEC-MRI, DWI method gives information of water protons as endogenous contrast to assess diffusivity and tissue microstructure in tumor, while IVIM can obtain multiple quantitative parameters, which can noninvasively separate pure molecular diffusion and capillary microcirculation perfusion. Petrillo et al. demonstrated that DWI showed higher AUC (0.81) than conventional T2WI (0.76) for assessing response to neoadjuvant therapy in locally advanced rectal cancer (25). IVIM-DWI is also reported to be applied to diagnosis of several tumors. Zhang et al. reported in a retrospective study that *D* value of IVIM-DWI could be used for prediction of result of concurrent chemoradiotherapy, with AUCs of 0.987 and 0.984 for training and test groups, respectively (30). In an animal study, it was found that *ADC* values were remarkably higher in pancreatic cancer mice treated with gemcitabine (31). Besides, it was found DCE-MRI combined with IVIM-DWI could enhance the diagnostic efficacy of ductal carcinoma in situ, in which the AUCs of *K_trans_*, *K_ep_*, *D* and their combination were 0.936, 0.902, 0.860, and 0.976, respectively (32). However, up to now, no study reported IVIM-DWI in osteosarcoma after neoadjuvant chemotherapy. In our study, we observed that *D*, *ADC* and *f* values of IVIM-DWI were markedly higher in CR/PR patients compared with SD or PD patients, which were negatively correlated with ALP and LDH. Besides, we also found *D* value after two treatment cycles showed good predictive value for PD. In an early study, T2WI was found to be able to predict the chemotherapy response, with AUC=0.70 in osteosarcoma (14). In our research, we found that using *K_ep_* value in DCE-MRI, the prediction of PD could achieve sensitivity 70.00% and specificity 92.31% with AUC 0.911, while *D* value of IVIM-DWI could also achieve the sensitivity 85.00% and specificity 81.12% with AUC 0.880 for prediction of PD, indicating that DCE-MRI and IVIM-DWI might be better than the conventional T2WI for chemotherapy response prediction.

However, we didn’t observe apparent difference for DCE-MRI and IVIM-DWI for prediction of treatment efficacy after neoadjuvant chemotherapy in osteosarcoma patients. Additionally, we found that values of *D*, *K^trans^* and *K_ep_* were all risk factors for PD after neoadjuvant chemotherapy. All these results indicated that both DCE-MRI and IVIM-DWI could effectively predict the treatment efficacy of osteosarcoma patients after neoadjuvant chemotherapy.

## Limitation

The study also has some limitations. First, the sample size is small and this is a single-center study. Secondly, the long-term clinical prognosis of the osteosarcoma patients is not clear and whether DCE-MRI and IVIM-DWI could also predict the patients’ prognosis is not investigated.

## Conclusion

In summary, we demonstrated that DCE-MRI and IVIM-DWI could be used to predict the clinical outcomes of osteosarcoma patients with neoadjuvant chemotherapy. The change of imaging parameters was associated with the clinical outcomes and both *D* and *K_ep_* values after two treatment cycles showed potential predictive value for diagnosis of PD. This study could bring deeper insights for DCE-MRI and IVIM-DWI in prediction of clinical outcomes in osteosarcoma patients.

## Data availability statement

The raw data supporting the conclusions of this article will be made available by the authors, without undue reservation.

## Ethics statement

The studies involving human participants were reviewed and approved by ethical committee of Hunan Cancer Hospital and the Affiliated Cancer Hospital of Xiangya School of Medicine, Central South University. The patients/participants provided their written informed consent to participate in this study. Written informed consent was obtained from the individual(s) for the publication of any potentially identifiable images or data included in this article.

## Author contributions

XX and LW conceived the idea of the study. FZ and JJL analyzed the data. QL interpreted the results. XX, LW, and QL wrote the paper. JL and XY discussed the results and revised the manuscript. All authors contributed to the article and approved the submitted version.

## Funding

This study was supported by (C2019-079) Research Fund of Hunan Provincial Health Commission Department (C2019-079) and (2020JJ8092) Hunan Provincial Natural Science Foundation of China (2020JJ8092).

## Conflict of interest

The authors declare that the research was conducted in the absence of any commercial or financial relationships that could be construed as a potential conflict of interest.

## Publisher’s note

All claims expressed in this article are solely those of the authors and do not necessarily represent those of their affiliated organizations, or those of the publisher, the editors and the reviewers. Any product that may be evaluated in this article, or claim that may be made by its manufacturer, is not guaranteed or endorsed by the publisher.
